# Depressive symptoms are associated with poor glycemic control among women with type 2 diabetes mellitus

**DOI:** 10.1186/s13104-018-3141-z

**Published:** 2018-01-16

**Authors:** Carlos Góis, Tiago Antunes Duarte, Sofia Paulino, João Filipe Raposo, Isabel do Carmo, António Barbosa

**Affiliations:** 1 Serviço de Psiquiatria e Saúde Mental do Hospital de Santa Maria–Centro Hospitalar Lisboa Norte, Av. Prof. Egas Moniz, 1649-035 Lisbon, Portugal; 2 Faculdade de Medicina da Universidade de Lisboa, Avenida Professor Egas Moniz, 1649-028 Lisbon, Portugal; 3 Associação Protectora dos Diabéticos de Portugal (APDP), R. Rodrigo da Fonseca 1, 1250-189 Lisbon, Portugal; 4 NOVA Medical School-Faculdade de Ciências Médicas, Campo Mártires da Pátria 130, 1169-056 Lisbon, Portugal

**Keywords:** Type 2 diabetes mellitus, Depression, Glycemic control, Sex

## Abstract

**Objective:**

In patients with type 2 diabetes mellitus, depressive symptoms may be associated with metabolic deterioration. The impact of sex on this association is unclear. The aim of this study is to analyze the relationship between depression and metabolic control by sex. The data presented is the side product of the clinical investigation by Rui Duarte, MD, Treatment Response in Type 2 Diabetes Patients with Major Depression from 2007.

**Results:**

A sample of 628 outpatients with type 2 diabetes mellitus was taken from a specialized diabetes outpatient clinic. In a univariate analysis: women’s glycohemoglobin mean levels were 8.99% whereas men’s were 8.41% and the difference was statistically significant. The proportion of women (34.3%) with pathological levels of depression (Hospital Anxiety Depression Scale score ≥ 8) was significantly higher than men’s (15.2%). A linear regression analysis performed by sex and controlling for demographic, clinical and psychological variables, showed poorer metabolic control in women with depressive symptoms. No association was observed in men. These results support depression as a predictor for poor metabolic control in women and the need for detecting depressive symptoms when glycemic levels deteriorate.

## Introduction

Comorbidity between diabetes and depression is common [1] and sex differences in type 2 diabetes mellitus (T2DM) have been reported frequently [2].

A recent prevalence study of the Portuguese population demonstrated that the proportion of undiagnosed T2DM patients is 43.6% with a point prevalence of 11.7% with a significant difference between men (14.2%) and women (9.5%) [3]. Another recent epidemiological survey found a prevalence of depression disorders in Portugal around 7.9%, women being at a higher risk of developing depression than men (OR = 2.30) [4]. There are no studies reporting the prevalence of diabetes and depression comorbidity in the Portuguese population.

Although estrogens lower the risk of coronary heart disease, diabetes eliminates that protective effect on women [5]. Estrogen replacement therapy showed a preventive effect on T2DM incidence in postmenopausal women [6] and was also associated with a decrease in depressive symptoms in perimenopausal women [7].

Negative coping strategies such as resignation, protest and isolation, were reported to be more prevalent in women with T2DM when compared to men [8]. Women also presented more difficulty on diet management and self-monitoring of blood glucose. They experienced their disease as an unforeseeable setback [9, 10] thus being less likely to reach glycemic goals with insulin treatment [11].

In women without diabetes, depressive symptoms are associated with a higher risk for metabolic syndrome. A greater waist circumference, higher fasting blood glucose, higher diastolic blood pressure, lower HDL-cholesterol and higher salivary cortisol were observed in women with depressive symptoms. No similar pattern was observed in men [12–14].

The few studies evaluating depressive symptoms by sex as associated with poorer glycemic control in T2DM showed conflicting results [15–18]. The finding of a sex difference would translate into better-adapted approaches, such as emphasizing the regular screening for depression in women with diabetes [19].

The present study aimed at detecting whether the presence of depressive symptoms was associated with poor glycemic control according to sex in a population with T2DM from a Portuguese diabetes clinic.

## Main text

### Methods

#### Patients

A screening protocol to detect depression and anxiety states in patients with T2DM was undertaken at the Portuguese Diabetes Association (APDP) and included in a multicenter screening study whose results were published elsewhere [20]. This protocol aimed to provide and evaluate specific clinical care for the comorbidity T2DM and depression. Inclusion criteria were age between 18 and 65 years; at least 6 months of T2DM diagnosis (type 1 diabetes (T1DM) was excluded); at least a 3 months’ follow-up at APDP; regular hemoglobin A1c (HbA1c) testing; and ability to understand and fill up questionnaires.

Portugal is a small southern European country with 10 million inhabitants. APDP is a private social solidarity institution, specialized in outpatient diabetes treating, founded in 1926 in Lisbon, and to where several general practitioners from southern Portugal sent patients regularly. More than 15,000 persons with T2DM and 4,000 with T1DM are yearly followed up at APDP. It also provides regular training on diabetes and patient education to nurses and medical doctors.

#### Methods

While waiting for their diabetes consultation, patients were approached and selected by trained clinical psychologists taking into account the inclusion criteria. Eligible patients were consecutively invited to participate and those who accepted were given a brief explanation about the negative influence of the comorbidity between diabetes and depression on health outcomes. Socio-demographic and clinical data, namely body mass index (BMI), HbA1c level, diabetes type, therapy and antidepressant medication, were collected. Chronic complications (retinopathy, nephropathy, neuropathy and cardiovascular) due to diabetes were identified from the patient’s medical history, physical findings and automated laboratory data.

Patients filled up the Portuguese version of the Hospital Anxiety Depression Scale (HADS) [21, 22]. HADS is a self-report Likert scale (0–3) used in previous studies for detection of depression and anxiety symptoms, consisting of 14 questions, 7 for anxiety and 7 for depression. Each sub-scale score ranges from 0 to 21, higher scores representing more severe symptoms. A score ≥ 8 on any of the sub-scales is considered pathological [22]. Only the depression symptoms sub-scale of HADS was used in the present study.

The HbA1c levels were determined at the APDP using an ion-exchange high-performance liquid chromatography in a BIO-RAD Variant II turbo kit. According to the manufacturing data, normal range is 4.9–6.2% (30–44 mmol/mol).

#### Statistical analysis

Results are presented as the mean ± standard deviation or as a percent as appropriate. Results between groups were compared using the Student’s t test or the Chi squared test. The Kolmogorov–Smirnov test was conducted to verify the normal distribution of the continuous variables. Prior to further analysis, non-normal distributed variables were log-transformed.

Linear regressions analyses were performed separately by sex with a model with all predictors entered concurrently. HbA1c was used as the dependent variable. Independent variables were the demographic and clinical characteristics, as well as depression and anxiety symptoms. To avoid multicollinearity, the only antidiabetic treatment variables considered were the combination therapy with oral antidiabetic agents (OAA) and insulin [23].

Results were considered statistically relevant when the *p*-value was < 0.05 using a 95% confidence interval. SPSS 21.0 was used to carry out statistical analysis (SPSS Inc., Chicago, Illinois).

### Results

#### Total sample

From July 2007 to July 2009 a total of 656 patients meeting the inclusion criteria were identified. From these, 15 refused to participate (response rate = 97.7%). Their demographic features were not different from those included in the study. Due to missing data, 13 patients were further eliminated. Therefore, this study comprised a clinic-based convenience sample of 628 persons with T2DM, men representing 52.4%, aged 57.69 ± 5.55 (range 36–65 years) (Table 1).

**Table 1:** Data displayed represent the mean values ± standard deviation

	Total	Men	Women	p-value
	N = 628	N = 329	N = 299	
Age (yrs)	57.69 ± 5.55	57.86 ± 5.34	57.50 ± 5.77	0.385
Education level (yrs)	6.57 ± 3.87	6.84 ± 3.93	6.27 ± 3.78	*0.035*
BMI (kg/m^2^)	29.99 ± 4.90	29.34 ± 4.55	30.70 ± 5.18	*0.001*
Years from diagnosis	13.89 ± 7.59	13.56 ± 7.59	14.26 ± 7.59	0.089
≥ 2 chr complications	162 (25.8)	102 (31.0)	60 (20.1)	*0.002*
≥ 1 comorbidities	84 (13.4)	37 (11.2)	47 (15.7)	0.102
OAA alone	259 (40.9)	157 (47.7)	100 (33.4)	*< 0.001*
Insulin alone	139 (22.1)	70 (21.3)	69 (23.1)	0.632
OAA and insulin	230 (36.6)	102 (31)	130 (43.5)	*0.001*
Antidepressant	64 (10.2)	17 (5.2)	47 (15.7)	*< 0.001*
HbA1c	8.69 ± 1.66	8.41 ± 1.48	8.99 ± 1.79	*< 0.001*
HbA1c < 7	84 (13.4)	55 (16.7)	29 (9.7)	*0.010*
HADS depression	5.16 ± 3.93	4.18 ± 3.29	6.25 ± 4.27	*< 0.001*
HADS depression ≥ 8	153 (24.4)	50 (15.2)	103 (34.4)	*< 0.001*

The respective percentage of the sample (global, men and women) is displayed between parentheses. Chi square and *Student’s t*-*tests* were used to compare each parameter between men and women. p-values are italic formatted when significance level is < *0.05.* Chronic complications: retinopathy, nephropathy, neuropathy and cardiovascular

*HADS* The Hospital Anxiety and Depression Scale, *BMI* body mass index, *OAA* oral antidiabetic agent, *HbA1c* hemoglobin A1c

Patients treated with insulin represented 58.7% of the sample, and 36.6% were also taking an OAA. The mean HbA1c level was 8.69% (50 mmol/mol) (SD = 1.66). Only 13.4% of the patients had an HbA1c level below 7% (53 mmol/mol). Depressive symptoms above the score threshold were present in 24.4%.

#### Sex differences

Women had significantly higher HbA1c values [mean = 8.99% (75 mmol/mol), SD = 1.79] than men [mean = 8.41% (69 mmol/mol), SD = 1.48] with a p-value = 0.0019. More men (16.7% men vs. 9.7% women, p = 0.010) reached significantly the therapeutic goal of HbA1c < 7% (53 mmol/mol). Women had significantly more depressive symptoms above the cut-off point (34.4% women vs. 15.2% men, p < 0.001). They are also three times more likely to take antidepressants than men (15.7% women vs. 5.2% men, significant p < 0.001). Men (31%) had more multiple (≥ 2) chronic complications than women (20.1%, significant p = 0.002). Women (43.5% vs. 31% men, p = 0.001) used significantly more combined therapy (OAA and insulin).

#### Depressive symptoms and other predictors of metabolic control by sex

Glycemic control was positively associated with depressive symptoms (standard beta = 0.12, significant p = 0.031) and negatively associated with comorbidities (standard beta = − 0.13, significant p = 0.020) in women. A positive significant association with higher HbA1c was found with combined treatment with OAA and insulin (standard beta = 0.16, p = 0.003) in men. A younger age was significantly associated with poorer glycemic control both in men (standard beta = − 0.24, p = < 0.001) and women (standard beta = − 0.19, p = 0.002) (Table 2).

**Table 2:** Multiple linear regressions analyses: associations of glycemic control and demographic, clinical and psychometric variables

	Men			Women		
	Beta-S	t	p-value	Beta-S	t	p-value
Age (yrs)	− 0.24	− 4.28	*0.001*	− 0.19	− 3.20	*0.002*
Education level (yrs)	− 0.03	− 0.62	0.534	− 0.08	− 1.38	0.170
BMI (kg/m^2^)	− 0.07	− 1.32	0.187	− 0.01	0.13	0.896
Years from diagnosis	0.07	1.11	0.268	0.04	0.60	0.550
≥ 2 chr complications	0.11	1.97	0.050	0.02	0.40	0.687
≥ 1 comorbidities	0.10	1.90	0.059	− 0.13	− 2.33	*0.020*
OAA and insulin	0.16	3.14	*0.003*	0.10	1.75	0.081
Antidepressant	0.02	0.86	0.776	− 0.07	− 1.17	0.241
HADS depression	− 0.01	− 0.11	0.985	0.12	1.77	*0.031*
Adjusted R^2^	0.079			0.065		
F-change	4.119			3.289		
p-value (F-change)	< 0.001			0.001		

Beta-S: Beta standard regression coefficients. Statistically significant results with *p*-values < 0.05 are highlighted in italics. Chronic complications: retinopathy, nephropathy, neuropathy and cardiovascular

*HADS* The Hospital Anxiety and Depression Scale, *BMI* body mass index, *OAA* oral antidiabetic agent

### Discussion

This cross-sectional study evaluated the association of depressive symptoms and glycemic control by sex, adjusting for possible confounders, in a sample of patients with T2DM.

Globally participants had extremely poor glycemic control. The relatively low education level of the sample could account for this result. In a study by Mezuk et al. [24], subjects with major depressive disorder and low educational attainment had a higher risk of T2DM. However, in our study, neither in men nor in women was detected any association between education level and glycemic control. As socioeconomic status usually encompasses educational attainment and can be associated with suboptimal preventive care and unhealthy lifestyle, if we had evaluated other features like income maybe we would have found an association with poorer glycemic control [25].

In our sample, women had poorer glycemic control than men as reported in previous studies [9]. Nonetheless, other studies describe similar HbA1c values in T2DM patients between sexes [9, 12, 15].

In our study, a statistically significant association was found in women between depressive symptoms and poor glycemic control, adjusting for age and education level, diabetes duration, chronic complications, comorbidities, combined antidiabetic therapy and antidepressant use. No similar association was found for men.

Combined therapy was more often used in women, but was independently associated with poor glycemic control only in men. These findings could suggest that women were more responsive to combined treatment although their poor glycemic control.

Women were three times more likely to take antidepressants than men, but only half (15.7%) of the women diagnosed with depression were taking antidepressants. Some studies suggest that half of the T2DM patients are not accurately diagnosed for depressive symptoms [17, 26]. In a study by Katon et al., only a third of the patients with depression had been adequately treated with an anti-depressant for at least 3 months [17].

Prevalence of undiagnosed depression in both sexes may arise at least partly from the effects of depression on how people perceive their health. Li et al. found that patients who reported poor or fair health had 2.8-fold greater likelihood of having undiagnosed depression [26].

Moreover, undiagnosed depression may be indirectly attributable to inability to correctly identify cases of depression as a function of sex. In a study based on a large British survey, men were more likely than women to misidentify depression in other men [27]. Beliefs such as hegemonic masculinity may affect men ability to self-report depression, leading them to report less symptoms overall than women. Nevertheless, one study found that men with higher adherence to masculine norms report more symptoms on measures of depression [28]. Another study showed that more than 20% of men who described themselves as depressed would not receive a diagnosis of depression by current criteria standards [29] suggesting the need for discussing traditional depression symptoms in what concern men.

In our study, similar to others results [15, 17, 18], men were less than half as likely to be depressed as women (34.4% women vs. 15.2% men, p < 0.001). The lower prevalence of depression in men may be attributable to underdiagnosis but we do not have any measure to declare or refute it.

### Conclusion

Our study suggests that it may be clinically relevant to conduct a systematic screening for depressive symptoms in T2DM patients. Women who are under an optimized antidiabetic therapy and still have HbA1c values above the control cut-off point need to be systematically checked for depression. Treating depressive symptoms, especially in middle-aged women, enables better hyperglycemic control and avoids metabolic deterioration.

## Limitations

This study has some limitations. Firstly, potential biological or psychological processes, link depression and T2DM. Hypothalamic–pituitary–adrenal axis, the innate immune response and the autonomic nervous system, and the burden with diabetes and unhealthy lifestyle behaviors, such as diet, physical activity and smoking [30], were not evaluated in our study. Secondly, the cross-sectional nature of the study does not allow for causality inferences. Chronic hyperglycemia can also affect depressive symptoms rather than vice versa, namely by the increase of diabetes burden [31]. Only with a longitudinal design would it be possible to determine the associative direction between hyperglycemia and depressive symptoms. Thirdly, depressive symptoms were analyzed with a Likert scale, but a psychiatric interview remains the gold standard for depression diagnosis in clinical practice.

Lastly, this represents a convenience sample from a single diabetes clinic with a population with poor glycemic control and low education level. Our findings may or may not be generalizable to other settings or patient populations.

Nevertheless, the large sample size is one of the study’s strengths. As far as we know, this is the first report to analyze the relationship between depressive symptoms and glycemic control in a Portuguese population with T2DM.

## Abbreviations

T2DM: type 2 diabetes mellitus
T1DM: type 1 diabetes mellitus
HbA1c: hemoglobin A1c
APDP: Portuguese Diabetes Association
BMI: body mass index
HADS: Hospital Anxiety Depression Scale
OAA: oral antidiabetic agents
